# Esophageal Perforation Following Radiofrequency Catheter Ablation for Atrial Fibrillation: A Conservative Approach

**DOI:** 10.19102/icrm.2022.130904

**Published:** 2022-09-15

**Authors:** Timothy Richard Maher, João Vítor Ternes Rech, Caique Martins Pereira Ternes, Alexander dal Forno, André D’Avila

**Affiliations:** ^1^Harvard-Thorndike Arrhythmia Institute and Division of Cardiovascular Diseases, Beth Israel Deaconess Medical Center and Harvard Medical School, Boston, MA, USA; ^2^Cardiac Arrhythmia Service—Hospital SOS Cardio, Florianópolis, Santa Catarina, Brazil

**Keywords:** Atrial fibrillation, catheter ablation, conservative treatment, esophageal perforation

## Abstract

Esophageal perforation following radiofrequency catheter ablation of atrial fibrillation (AF) is a rare and potentially deadly complication. Here, we report a case with successful conservative management of esophageal perforation following AF ablation demonstrating how surgery is not always required in properly selected patients.

## Case presentation

A 74-year-old woman presented with dysphagia, odynophagia, and colicky abdominal pain radiating to the back approximately 1 week following a repeat pulmonary vein isolation ablation for recurrent atrial fibrillation (AF) using radiofrequency ablation (RFA). During the procedure, the left common pulmonary vein trunk was found to be reconnected, requiring lesions on the posterior wall near the esophagus to achieve re-isolation. Ablation lesions were delivered with an irrigated contact force–sensing ablation catheter at 35 W for 12 s with a force of approximately 10 g. An esophageal temperature monitoring probe was placed but was unable to be used during the ablation due to a monitor malfunction. There were no acute complications during the procedure, and the patient was prescribed a proton pump inhibitor with a planned course of twice daily for 2 weeks followed by once daily for 2 weeks. At the time of transfer, the patient was normotensive and afebrile with a temperature of 96.8°F; cardiac examination revealed no noteworthy abnormalities.

### Past medical history

The patient had a past history of diabetes mellitus, dyslipidemia, and recurrent paroxysmal AF refractory to anti-arrhythmic drugs.

### Investigations

**Figure 1:**
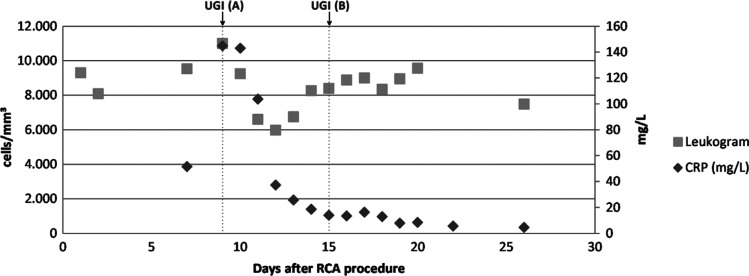
C-reactive protein level and leukogram results throughout the first 30 days of the conservative treatment of the esophageal perforation, showing a decrease of C-reactive protein between days 10–15.

**Figure 2:**
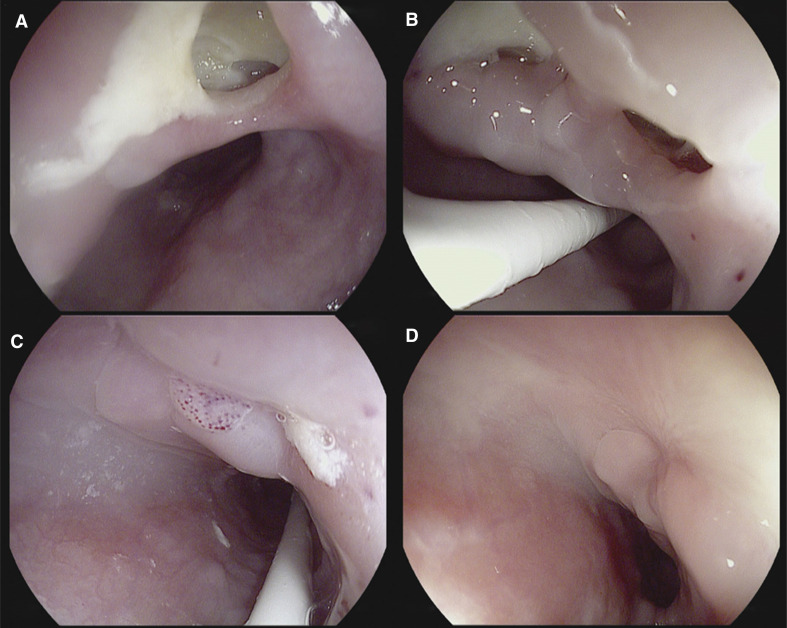
Esophagogastroduodenoscopy findings of **(A)** esophageal lesion perforation without fistula formation 8 days after catheter ablation, **(B)** injury containing fibrin on the 15th day after surgery, **(C)** damage area with granulation tissue, and **(D)** the previously damaged area healed.

Her laboratory tests were significant for a mildly elevated white blood cell count of 11,000 and C-reactive protein (CRP) level of nearly 60 mg/L **(Figure 1)**. An urgent chest computed tomography (CT) scan with intravenous (IV) contrast and esophagogastroduodenoscopy (EGD) were performed, which revealed an esophageal perforation located 13 in (33 cm) below the upper dental arch **(Figure 2A)** without atrial or pericardial involvement, which was characterized as a type 3A lesion according to the recently proposed Kansas City classification.

### Differential diagnosis

The differential diagnosis included esophageal erosion, esophageal perforation, atrioesophageal fistula (AEF), and post-ablation pericarditis.

## Management

In the intensive care unit (ICU), she was given nothing by mouth (NPO) and started on total parenteral nutrition, broad-spectrum IV antibiotics for 7 days, and IV proton pump inhibitor therapy. Post-ablation, anticoagulation was continued with IV unfractionated heparin. Based on the patient’s clinical stability and following interdisciplinary discussion between the surgical and cardiac electrophysiology teams, the decision was made to continue a conservative management approach with close observation and frequent reassessment of symptoms and hemodynamics. On day 7 after admission, CT and EGD were again performed, which showed 2 esophageal injuries **(Figure 2B)** with ulceration and permeating fibrin around the injury. A nasal enteric tube was introduced under direct visualization during the endoscopy. On day 8, the patient was tolerating parenteral nutrition, was afebrile and normotensive, and her CRP level had downtrended, so she was transferred from the ICU to a medical floor. At day 22, the patient had no worsening of her clinical condition and an additional repeat EGD showed complete healing of one of the esophageal injuries, a closure of about 50% of the previously damaged area, and the presence of granulation tissue **(Figure 2C)**. The patient remained hospitalized for further monitoring and patient preference. On inpatient day 46, EGD revealed complete healing of the esophageal injury **(Figure 2D)**, and the patient was discharged.

## Discussion

RFA for AF is a well-accepted treatment option to reduce symptomatic recurrences and improve quality of life in patients with both paroxysmal and persistent AF.^1^ RFA is more effective than anti-arrhythmic drugs for maintaining sinus rhythm, and although RFA is regarded as a safe procedure with very low rates of major complications (the incidence of death following ablation of AF has been reported as up to 4.2 in every 1,000 AF ablations), many intraoperative and postoperative complications, including stroke, cardiac tamponade, and esophageal injury, have been reported.^2^ Of note, the ablation parameters used on the posterior wall during this procedure (35 W for 10–12 s with approximately 10 g of force) are relatively modest and serve as a humbling reminder that serious esophageal injury can occur despite nominally low-energy delivery parameters, and other factors can increase the risk of injury, including left atrial wall thickness/fat content, distance to the anterior wall of the esophagus, stable catheter position, and reduced esophageal motility under general anesthesia.^3,4^ The presence of potential prior adhesions adhering the esophagus to the posterior pericardium as this was a repeat procedure is also possible.

Esophageal injury from AF ablation consists of a spectrum of lesions ranging from the relatively common and more benign esophageal erosion and ulceration to the rare and potentially catastrophic esophageal perforation and AEF.^5^ These latter complications can result in mediastinal infection, stroke, and death. In spite of a number of reports and observational studies that have been published on the topic of AEF, there is significantly less information about esophageal injuries without the formation of AEF, such as esophageal perforation following RFA of AF.^4^ Esophageal perforation is most commonly treated with early surgical management due to the high associated morbidity and mortality rates, but conservative (nonoperative) management is occasionally used in the properly selected patient. There are no established guidelines or randomized prospective clinical studies related to the management of esophageal perforations following RFA for AF, primarily due to the rarity of the complication. AEFs are associated with high rates of mortality (55%–80%), with best outcomes associated with aggressive early surgical repair (with a 33% mortality rate) compared to esophageal stenting (68% mortality) or conservative care (97% mortality).^6^ Esophageal perforations, while also commonly associated with high mortality rates (20%–33% with iatrogenic perforations), do have a body of literature dating back to 1965 supporting conservative/nonoperative care in the properly selected patient.^7,8^ Conservative treatment is recommended to include close observation in a critical care setting with cardiopulmonary monitoring; making the patient NPO; providing IV fluids, parenteral nutrition (during a prolonged course), and broad-spectrum IV antibiotics for 7–10 days; and reassessing with contrast esophagography in 7 days prior to challenging with oral intake.^8^ This patient had a protracted hospitalization out of an abundance of caution given the limited data on conservative management as well as the need for enteral feeding and patient preference given the high risk of complications. The length of hospitalization should balance patient stability, clinical trajectory, resource utilization, and patient-centered preferences. With close outpatient follow-up after both marked clinical and endoscopic improvement, some patients may be candidates for earlier discharge than this patient. In the surgical literature, the length of stay for esophageal perforation has a mean of 41 days.^7^

Commonly used criteria to select patients for conservative therapy include early diagnosis or delayed diagnosis with contained leak, perforation not in the abdomen, contained perforation in the mediastinum, content of the perforation draining back into the esophagus, the perforation does not involve neoplasm or obstruction of the esophagus, absence of sepsis, and presence of an experienced thoracic surgeon and contrast imaging at the hospital. Indications for urgent surgical intervention in these patients include any signs or symptoms of sepsis, respiratory failure, pneumothorax, or mediastinal emphysema.^8,9^ Mortality rates with conservative therapy can range as high as 38%, but, in properly selected patients, case series show mortality rates as low as 0%, particularly in the context of iatrogenic perforations, as they are associated with less extraluminal contamination. Alternatives to surgical and conservative therapy that have also been used successfully for treating esophageal perforations include esophageal stenting, fibrin glue injection, endoscopic vacuum therapy, and endoclipping via endoscopy, though care must be taken during endoscopy as insufflation can worsen a perforation.^8^ When fluid collections are discovered on repeat imaging, percutaneously placed drains should be used as an adjunct therapy.^10^

Yarlagadda et al. recently proposed a stratification of esophageal injuries from RFA called the Kansas City classification, which grades lesions as follows: type 1 lesions (erythema of the esophagus tissue), type 2A (superficial ulcer), type 2B (deep ulcer), type 3A lesions (esophageal perforation without fistulous communication with the atria), and type 3B lesions (AEF).^11^ In the systematic review of 4,473 patients used to derive the Kansas City classification, injuries up to type 2B lesions (without an esophageal perforation) were conservatively managed with proton pump inhibitors and, in some cases, hospital admission with NPO, IV broad-spectrum antibiotics, and intensive care. Of the 6 patients with type 3 lesions, 5 had type 3A lesions (similar to our patient in this report), among whom 4 recovered following esophageal stenting and 1 developed a pericardioesophageal fistula and died from sepsis following surgery. There was also 1 patient with a type 3B lesion, and this patient died.

Although there is still no consensus on the management of type 3A lesions, the few cases published **(Table 1)** have predominantly reported invasive management with either surgical or endoscopic intervention. In contrast, the patient presented in this case report was selected for conservative treatment with a successful outcome. The conservative approach was chosen because the patient had a late presentation and stable hemodynamics from an iatrogenic contained thoracic esophageal perforation with an otherwise normal esophagus and was able to be closely monitored by the electrophysiology, critical care, and thoracic surgery teams. Small, transmural, thoracic perforations with local, circumscribed extravasation of contrast and the NPO status of postoperative patients are favorable prognostic indicators for conservative management of esophageal perforations^10^ and should be also considered when deciding over a surgical or medical approach to esophageal perforations after RFA for AF. Also, while medical management should always be evaluated as a viable option, clinical deterioration, sepsis, or extension of the perforation should raise an alarm for the reconsideration of surgery.^8,10^

## Conclusions

There are no randomized controlled trials to guide evidence-based treatments of esophageal perforations after RFA for AF. We present a case of an esophageal perforation following RFA of AF that was successfully treated with a conservative approach, demonstrating medical management as a viable treatment strategy for the properly selected patient who is hemodynamically stable with a small transmural intrathoracic perforation and who can be closely monitored and frequently reassessed.

At 1 year of follow-up, our patient presented in a good condition and without any associated symptoms or complications.

## Figures and Tables

**Table 1:** Summary of Esophageal Perforation Cases

Author/Year Published	Year Published	No. of Patients	Time After Procedure	WBC Count on Admission	Symptoms	Urgent Treatment	Definitive Treatment	Complications
This report	2022	1	1 week	Leukocytosis (11,000/μL)	Dysphagia, odynophagia, and abdominal pain	Medical	Medical	None
Mitchell-Brown and McPherrin^12^	2018	1	2 weeks	—	Chest pain	Stent placement	Medical	—
Katz-Agranov and Nevah Rubin^13^	2017	1	7 days	—	Chest pain, dysphagia, odynophagia followed by hematemesis	Medical	Stent placement	Patient died soon after
Manouchehri et al.^14^	2014	1	2 days	Leukocytosis (19,400/μL)	Odynophagia, chest pain	Thoracotomy, patch with vascularized intercostal muscle flap	Stent placement	—
Dagres et al.^15^*	2006	1	8–28 days	Leukocytosis (15,460 ± 2,910/μL)	Fever, chest pain	—	Extensive surgical esophageal resection	—
Bunch et al.^16^	2006	1	2 weeks	Leukocytosis (13,000/μL)	Chest pain, fever, solid food dysphagia	Stent placement	Medical	Stent migration correction, pain control required due to discomfort and pain after stent placement

*Abbreviation:* WBC, white blood cell. *Dagres et al. reported 4 patients, but 3 had intraoperative ablations, and these were not clearly distinguished from percutaneous procedures.
